# Executive Functions Can Be Improved in Preschoolers Through Systematic Playing in Educational Settings: Evidence From a Longitudinal Study

**DOI:** 10.3389/fpsyg.2019.02024

**Published:** 2019-09-03

**Authors:** Ricardo Rosas, Victoria Espinoza, Felipe Porflitt, Francisco Ceric

**Affiliations:** ^1^Escuela de Psicología Centro de Justicia Educacional, Pontificia Universidad Católica de Chile, Santiago, Chile; ^2^Facultad de Educación, Pontificia Universidad Católica de Chile, Santiago, Chile; ^3^Facultad de Psicología, Universidad del Desarrollo, Santiago, Chile

**Keywords:** executive functions, intervention program, preschool, play, inhibitory control, working memory, cognitive flexibility

## Abstract

This study aimed to test the impact of play on the development of executive functions (EFs) in preschoolers. Thirty-two games were designed to be collectively played in groups by 70 children, in their regular classes. The games were specifically designed to promote the development of the three components of EFs: inhibition (behavioral or cognitive), working memory, and cognitive flexibility. The games focused on each function were of three types: playground games, expression games, and classroom games. Sixty 45 min play sessions were held on consecutive days for 3 months, always in the first period. The sessions were guided by two members of the research team, assisted by the four teachers of the participating classes. The intervention was carried out in two highly socially vulnerable schools in the city of Santiago de Chile. Four classes were studied in total: two experimental groups and two controls. The classes were selected using a questionnaire on teacher-student interaction quality and an age homogeneity criterion. EFs were evaluated using the Hearts and Flowers task at three points: before the intervention (T1), immediately after the end of the intervention (T2), and 8 months after the end of the intervention (T3). The results show a significant difference in the growth of EFs by comparing the experimental and control groups (*p* = 0.04) between T1 and T3. They also reveal a strong correlation between EFs measures at T1 and mathematics performance at T3. These results are discussed within the context of the guidelines proposed by Diamond and Ling (2016) and Barnett (2011) regarding what an EFs promotion program needs to be considered effective and high quality. The program presented in this study meets most of the requisites mentioned by the authors, which proves that following these guidelines guarantees a high probability of success.

## Introduction

Executive functions are psychological processes that enable us to plan and monitor our actions. They involve our ability to keep our thoughts, actions, and emotions under conscious control (Zelazo and Müller, 2011). Three components of EFs are commonly distinguished: inhibitory control, working memory, and cognitive flexibility (Diamond, 2013; Snyder et al., 2015; Bardikoff and Sabbagh, 2017).

Inhibitory control allows us to consciously direct our attention to stimuli that will enable us to conduct a task. This cognitive function permits us to avoid thoughts, behaviors, or emotions unsuited to the demands of a given situation (Friedman and Miyake, 2004; Diamond, 2013). Specifically, control of one’s emotions, thoughts, and affects has been labeled as cognitive inhibition, whereas control exerted over one’s actions is known as behavioral inhibition (Lampe et al., 2007).

Working memory means the ability to operate with mental representations, that is, to remember and use information simultaneously. It is a limited capacity that increases with age. Working memory is essential to establishing connections between prior knowledge and new information (Carriedo et al., 2016), generating non-evident associations, and understanding expressions of various types (Diamond, 2012, 2013).

Lastly, cognitive flexibility is an ability that enables us to adjust to the demands posed by the environment in an efficient manner (Miller and Cohen, 2001) by creating alternative ways of solving problems from multiple perspectives (Diamond, 2012), shifting our attention, or changing our strategies according to stimuli (McGowan et al., 2018). Cognitive flexibility is a relevant socio-affective component since it involves not only adopting divergent strategies to solve one’s problems but also understanding the approaches used by others. In brief, it is both an affective and a cognitive function that is closely linked to creativity (Diamond, 2014; Santa Cruz and Rosas, 2017).

### Development of EFs

Executive functions involve a long developmental process that begins during the perinatal period, sharply increases in the preschool stage, and reaches its apex during adolescence (Shonkoff et al., 2011). This process is supported by the development of the prefrontal cortex (Lezak et al., 2012), a brain area that hosts higher psychological functions, which are key to achieving adequate social and cognitive functioning (Rueda et al., 2011; Wiebe et al., 2011; Posner, 2012).

Although the growth of EFs follows a common trend, it has been proposed that their components do not develop as a unit; rather, each individual EFs follows its own trajectory (Diamond, 2006). Yet authors have suggested that these trajectories operate in tandem, with certain factors forming the basis for the development of others. Inhibitory control has been described as laying the groundwork for the development of EFs, followed by working memory and cognitive flexibility (Anderson et al., 2001). Thus, the development of inhibitory control has been reported to make it possible for working memory to grow, with both enabling individuals to increase their cognitive flexibility skills.

It has been proposed that although all the components of EFs start developing in the first years of life, their individual development trajectories differ. Inhibitory control has been described as having a very steep developmental slope between 3 and 5 years of age, which becomes weaker from age 5 onward, sharply declines after age 8, and becomes stable around age 12. Working memory, for its part, has a more gradual development trajectory, with a linear increase being observed between 4 and 14 years of age and stabilization being reached in adolescence. Lastly, research suggests that cognitive flexibility also gradually develops in childhood and reaches its peak around age 15 (Best et al., 2009; Best and Miller, 2010).

The development of the components of EFs allows reasoning, problem-solving, and planning to manifest themselves (Diamond, 2013, 2016; Baggetta and Alexander, 2016). These higher psychological processes are essential when confronting the demands of school life and those that entail adult life.

### Why Play Is Important for the Development of EFs at Preschool Age

As noted above, the components of EFs develop at a much faster rate in the preschool stage. It is precisely at this stage that children are first exposed to schooling, where environmental demands are key to promoting the early development of EFs (Rothbart and Posner, 2006; Garon et al., 2008), which in turn help improve school learning (Rimm-Kaufman et al., 2009).

Preschool education has been described as a space that makes it possible to strengthen the development of skills and knowledge that children require to adequately perform at later stages of school education (Pianta et al., 2009). At this stage, children are expected to develop the skills that lay the groundwork for the acquisition of reading and mathematical skills (Whitehurst and Lonigan, 1998; Espy and Cwik, 2004), which are modulated by the development of EFs. In addition, children are expected to improve their skills needed to develop adaptive behaviors that will enable them to meet the demands of the school system (Blair, 2002). These include self-regulation and social competence, both of which allow students to be motivated, focused, and persevering when dealing with tasks in order to complete them successfully (Kochanska et al., 2000). These skills are also grounded in the development of EFs, inasmuch as they allow thought and behavior to become organized while inhibiting automatic responses to attractive stimuli and privileging more self-regulated behaviors (Kochanska et al., 2001; Bierman et al., 2008).

However, not all educational environments promote the development of EFs equally. There is evidence that shows that stress and poor fitness negatively affect the functioning of the prefrontal cortex, and thus of EFs (Diamond and Lee, 2011). In this context, the educational programs that have proven to be most successful in developing EFs share two key characteristics: (1) they do not expect children to remain seated for long periods since this is not in line with their stage of development, generating tension between teachers and students and increasing children’s fear of school, and (2) they tend to reduce stress in the classroom, encouraging enjoyment, self-confidence, and the development of social ties.

Ludic environments could be spaces that foster the development of EFs if they take into account the needs of preschoolers and implement activities that promote the improvement of students’ physical condition. Play-based interventions have been shown to be effective when they increase the development of skills associated with divergent thinking, problem-solving, and life satisfaction (Moore and Russ, 2008).

Various types of games can support the development of EFs. There is evidence linking the use of video games designed to foster visual working memory skills (Thorell et al., 2009) and attention (Tahiroglu et al., 2010; Anderson and Bavelier, 2011) with better EFs development in preschoolers. In addition, authors have reported that EFs improve as a consequence of engaging in games based on aerobic exercises (Davis et al., 2011) and sports such as karate (Lakes and Hoyt, 2004). It has also been suggested that role-playing activities are tools that contribute to the development of emotional regulation and language, both of which are regarded as precursors of EFs (Fantuzzo et al., 2004). Other authors have reported that children’s performance improves when EFs are evaluated through play (Rosas et al., 2015).

Play makes it possible to reduce anxiety, which increases motivation and provides further chances to try out solutions and practice with no real consequences (Cadavid-Ruiz et al., 2014). Also, given that play is the predominant activity at the preschool stage, it can be regarded as a mediator that promotes children’s cognitive development (Vygotsky, 2001). In short, play is considered to be one of the key activities in children’s life at the preschool stage (Duncan and Tarulli, 2003).

### Successful Play Intervention Programs for the Development of EFs in Preschoolers

The literature describes a variety of successful EFs training initiatives. Authors have also referred to the necessary conditions for EFs interventions to succeed.

Traverso et al. (2015) conducted an intervention focused on the development of working memory, inhibitory control, and cognitive flexibility with 75 children aged 5. Twelve play sessions lasting 30 min each were conducted for over 1 month at the educational center that these children attended. The children were divided into groups of five and performed tasks that required progressive levels of inhibitory control, working memory, and cognitive flexibility. The results indicate that the children who took part in the intervention performed better in tasks involving simple EFs as well as in others requiring complex EFs. To analyze the effectiveness of the intervention, the authors compared the students’ performance in the tasks presented. Significant differences were observed in most tasks, controlling for initial performance. The children in the experimental group performed significantly better in inhibition tasks (delay task, gift wrap task time, circle drawing task, preschool matching familiar figure task, arrow flanker task), working memory tasks (backward word span, keep track task), and cognitive flexibility tasks (point accuracy task). This suggests that the children who participated in the training sessions performed better than those in the control group.

Specifically for EFs, Diamond et al. (2007) noted that children trained with “Tools of the Mind”, which is a research-based model that implies the implementation of a preschool curriculum focused on the development of cognitive, social-emotional, self-regulatory and foundational academic skills of children, perform better than their untrained peers in overall EFs, with minor effects in tests with low EFs requirements and major effects in tests with greater EFs demands, which benefit from more inhibitory control.

In the same way, Goldin et al. (2014) assessed several aspects of EFs (working memory, inhibitory control, flexibility, and planning) and school grades (language and mathematics), comparing children who used a computer program aligned with the Argentinian school curriculum and designed to train these variables (7 h of training in total over 10 weeks). Children in the experimental group played three adaptive computer games focused on training EFs, and children in the control group played games that require similar motor responses but were less demanding cognitively. All children played during school time, one game per 15-min session. The authors presented evidence that showed that children who received this training exhibited improvements in working memory as measured by the Attention Network Test (Rueda and Posner, 2013) and in inhibition and cognitive flexibility as measured by the Hearts and Flowers task (Davidson et al., 2006).

Another example of a play-based intervention was reported by Hermida et al. (2015), who generated a program that involved a longer training period: twice a week for 16 weeks. These authors carefully designed an intervention in which each activity had to meet the following conditions: (a) must be based on an aspect of the official school curriculum of the city of Buenos Aires; (b) must be structured as a game; (c) must require an increasing level of executive functioning; (d) must have three chronological stages (i.e., teacher-provided planning, execution of the planned activity and discussion of the activity with the children, and integration, with the children evaluating the plan and the strategies needed to implement it); (e) must be novel and different from previously introduced games, and; (f) must target an EFs clearly identified by the teachers, who had to be aware of which specific part of the activity trained EFs selected. They assessed the children in a variety of cognitive tasks at the beginning and after finishing the intervention. Also, they collected the grades of the children of both groups the year after the intervention.

Results for cognitive variables show that only differences in favor of the experimental over the control group exist, in the general measure of the Attention Network Test (Rueda and Posner, 2013) and in the selection of four blocks in the Corsi block-tapping test (Kessels et al., 2010). However, since these represent only two dependent variables out of 20, the authors suggested that the results cannot be attributed to the intervention. However, the experimental group showed significantly better performance in both language and math grades one year after the intervention, when comparing the experimental and control groups. They also compared these results with an external control group with similar demographic characteristics (not part of the study) and found similar results, suggesting a lasting effect of the training over the general school outcomes of the children. The authors noted that the rejection of the main hypothesis (posttest cognitive advantage for the intervention group) could be due to several factors: (1) the use of a test battery that might have been suboptimal for interventions of this type, (2) the time that the intervention lasted and the intensity of the activities (32 weeks, two games per week), and/or (3) the composition of the sample since ethical considerations demanded that an experimental design be avoided: the unit of analysis included whole classes (each with its own dynamics) participating in the intervention program, not individual participants.

Finally, although it is not totally based on play, the intervention program of Röthlisberger et al. (2011) is particularly relevant to the present work because of their strong similarities. The authors developed a small group intervention in EFs for a total of 33 prekindergarten and 30 kindergarten children, for 30 min in consecutive schooldays for a total of 6 weeks. A total of 19 tasks that would promote EFs were designed, specifically for working memory, interference control, and cognitive flexibility. The tasks were presented 2 days a week by a research team member and the remaining 3 days by a regular teacher. Group sizes for both the intervention and the control groups varied between 3 and 11 children. All the sessions, which lasted for about 30 min, included whole group activities, small group ones, and individual ones. Although not all tasks were games, all of them were highly motivating to the children. The three EFs components were assessed separately: interference control, by an adaptation of the Simpler Flanker Task (Roebers and Kauer, 2009); working memory, by an adaptation of the Complex Span Task (Daneman and Carpenter, 1983); and flexibility by an adaptation of the Flanker Task from Diamond et al. (2007).

The results show significant training effects for working memory and flexibility in the prekindergarten group and for interference control only in the kindergarten group.

One important issue that arises from these studies is that they can all demonstrate significant effects over at least one of the EFs components. Nevertheless, none of them give a sound theoretically grounded explanation as to why their particular programs have a specific impact over only some of the EFs components. We believe that these results show that at preschool age, EFs are not so clearly differentiated and thus cannot be reliably measured separately. In the present project, we will therefore use only one global measure of EFs, although we will differentiate the EFs components to be trained in the intervention program.

### A Framework for the Design of Successful EFs Enhancement Intervention Programs

Diamond and Ling (2016) analyzed several studies on interventions that successfully improved EFs development, drawing a number of conclusions about the characteristics of these initiatives. The following is a brief description of the authors’ conclusions. (1) Although training appears to have a high degree of transference, it tends to be strongly associated with the cognitive function trained. For this reason, to avoid predictability, the authors suggest developing varied tasks that require the use of multiple cognitive skills. (2) Practice time is important, as programs that include more weekly sessions and are applied over a longer period have better outcomes. (3) The way in which the activity is presented and conducted can also influence the program’s outcomes: it has been observed that when a program is administered by more committed people, more benefits are observed. (4) EFs must be constantly challenged. (5) Individuals with lower levels of EFs development benefit more from programs of this type, with potential differences being due to age, socioeconomic status (SES), or the presence of disorders. (6) The impact of programs fades over time. (7) Differences that can be attributed to the impact of a program are often observed only in the most cognitively demanding tasks. (8) Physical training without a cognitive component has little impact on EFs development. (9) It is necessary to analyze the largest number of intervening factors possible to determine whether the results obtained are due to the program or to other factors related to it. For instance, benefits may be due to the type of mediation rather than to the cognitive tasks proposed; alternatively, gains could be mediated by the impact of the program on other factors such as stress reduction.

Also, extending the effects of interventions to other cognitive aspects, evidence shows that cognitive gains appear to be small initially, but longitudinal studies indicate that they increase as children grow up (Nix, 2003) and that effective interventions tend to be part of low-scale, high-quality programs (Schweinhart et al., 2005). Thus, program quality should be ensured, considering the aspects that have shown to be key: clarity regarding what the program provides, who its target audience is, and what wider educational, social, and economic contexts it encompasses (Barnett, 2004). These three factors become especially relevant considering that low-quality programs do not produce good results and that significant long-term effects are observed only when programs protect their high quality (Barnett and Masse, 2007). In consequence, authors recommend that interventions be implemented in both developed and developing countries if good quality can be ensured (Barnett, 2011).

In brief, research suggests that intervention programs, both play-based and not play-based, aimed at promoting EFs development in preschoolers must meet certain requirements in order to succeed. The present study was designed considering the main findings derived from interventions that have successfully improved EFs development in preschoolers, based on play activities in a natural context.

## Materials and Methods

### Participants

A total of 70 preschool monolingual Chilean children, out of whom 57% were boys and 43% were girls, participated in the research program. The average age was 68.42 months (*SD* = 3.48). The experimental group was composed of 37 children (*M* = 68.24 months; *SD* = 3.39), and the control group consisted of 33 children (*M* = 68.61 months; *SD* = 3.46). Both groups had the same proportion of boys and girls as the complete group.

The participants were recruited from two schools located in vulnerable areas in the city of Santiago de Chile. All children belonged to middle-low SES families. The SES classification is determined by the Quality Agency of Education of Chile and is constructed by considering the educational level of both parents, the total monthly economic income of the household, and the student vulnerability index. This index is calculated by determining the percentage of school students who are in an extreme poverty situation or who are at risk of school failure. The first three indicators are obtained through a survey given to the parents of the students in a national assessment, while the fourth is obtained from data collected by the National Board of School Aid and Scholarships of Chile. A middle-low SES school category means that its community includes families whose parents on average have 10 years of formal education with an average monthly family income of around US$ 358, and with 72% of the students in a vulnerable situation.

All the children attended the second transition level at the start of the intervention program. This level precedes the first grade of primary education. In Chile, there are six levels of preschool education. The first two levels correspond to nursery (from 84 days old to 2 years old), the next two are middle-age levels (from 2 to 4 years old), and the last ones correspond to transitions levels, including prekindergarten (5 years old) and kindergarten (until 6 years old).

### Procedure

Four different classes were selected, one experimental class from each school and two control classes from one of the schools. The classes were randomly assigned to each condition.

One of the schools had four classes in Kindergarten, and the other school had two. We included in the sample three classes from the first school and one from the second because the other classes did not meet the inclusion criteria. The first criterion was age, which was controlled by selecting classes with at least a median age of 68 months. This decision was taken because in a previous analysis we observed that the reliability of the EFs measurement was weak for the youngest part of the sample. The second inclusion criterion was the quality of instructional interactions between educators and children, measured by CLASS Pre-K^®^ (Pianta et al., 2008). This test shows the quality of interaction between educators and children in the classroom through three main indicators (emotional support, class organization, and instructional support). The research team hired a certified professional in CLASS Pre-K. Assessment was made through six different observation periods of 20 min each in three different days. Each observation was qualified on a 7-point scale. The quality of interactions can be high (6 to 7 points), middle (3 to 5 points) or low (1 to 2 points). To be included in the sample, classes needed to exhibit the high or middle quality of interactions in all the domains. One of the classes presented low-quality interactions in the instructional domain of the instrument, which determined its exclusion from the experiment.

The participants had never been included in any other cognitive intervention programs and received no incentives for taking part in the study. The parents signed an informed consent form to authorize their children to participate in the study, and these children gave their verbal assent before the beginning of each evaluation. The study was approved by the Vicerrectoría de Investigación (VRI; Vice President’s Office for Research) of the Pontificia Universidad Católica de Chile through the Ethics Committee of the Faculty of Social Sciences of the School of Psychology, thus meeting international norms for social science research.

Games were played with the complete class, but only children who were authorized by their parents and who voluntarily agreed to participate were assessed and included in the research sample. Classes had about 35 children each.

### Measures

**FIGURE 1:**
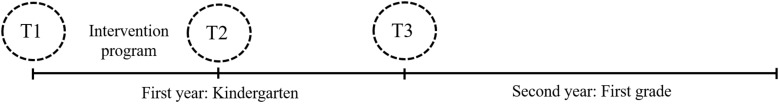
Distribution of time during the research process.

The EFs of the children from all groups were assessed at three different times. The first assessment was made before the implementation of the program, when the students were starting kindergarten (T1). Then the participants were assessed using the same test after finishing the intervention, when they were in the middle of kindergarten (T2). And then they were assessed 8 months later, when they were starting first grade (T3). Academic performance was assessed at the last evaluation point. Figure 1 shows the assessment and intervention times.

**FIGURE 2:**
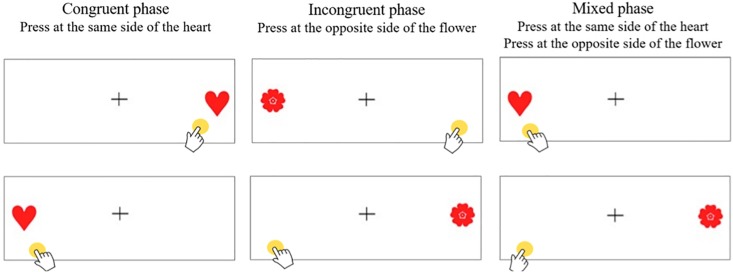
Examples of the Hearts and Flowers test items at different phases.

The Hearts and Flowers task (Wright and Diamond, 2014) was used as a general measure of EFs in all the three measures. Reliability of this test is not reported by the authors, but an adapted version with a Chilean sample, obtained a Cronbachs α = 0.83 (Rosas et al., 2019). In this task, participants are required to use a tablet device to respond to congruent and incongruent visual stimuli within a set time limit. The task comprises three phases. The first phase is the congruent phase, in which the child must touch the same part of the screen when a stimulus (heart) appears 12 times. The second phase has an incongruent stimulus, in which the participant must touch the opposite side of the screen when the stimulus (flower) appears also 12 times. The third phase is the mixed phase, in which both congruent and incongruent stimuli are randomly presented 33 times. In all the phases, the stimuli are shown for 750 ms, and then disappear for 1 s (response time), and then another stimulus is presented. The total number of correct answers in phase 3 is used as an indicator of EFs performance. Figure 2 shows the three phases of the test.

Academic performance in the language area was evaluated through phonological awareness and word reading skills. Phonological awareness was measured using the rhyme detection subtest of the Woodcock-Muñoz battery (Cronbach α = 0.98) (Muñoz-Sandoval et al., 2005). In the rhyme test, participants must select the option that ends with the same sound as the target word. Word reading is assessed using the letter and word identification test of the Woodcock-Muñoz battery (Cronbach α = 0.98) (Muñoz-Sandoval et al., 2005), in which participants read words and receive a score according to their reading accuracy. The complexity of this test gradually increases based on the syllabic structure, length, and frequency of the words used.

Performance in the mathematics area was assessed using problem-solving and counting skills. Problem-solving skills are assessed using the problem-solving scale of the Woodcock-Muñoz battery (Cronbach α = 0.95) (Muñoz-Sandoval et al., 2005), in which participants must quickly solve addition, subtraction, and multiplication problems within 3 min. Counting skills are assessed through an adaptation of the paradigm proposed by Koponen et al. (2012) (Cronbach α = 0.72). The task has two parts: forward counting (from 1 to 51, from 18 to 25, and from 6 to 13) and backward counting (from 33 to 17, from 23 to 19, from 12 to 7, and from 23 to 1).

It is important to note that because of the extreme SES homogeneity of the Chilean educational system, IQ also tends to be extremely homogeneous (Rosas and Santa Cruz, 2013) and therefore was not considered as a relevant covariable in the present study. As the authors show in the cited works, even small increments in parents’ copayment for public school education determine causal differences in the children’s cognitive outcomes. There is an almost perfect linear relationship between SES and cognitive outcome in the Chilean educational system (Rosas and Santa Cruz, 2014).

Trained psychologists (different as the game mediators) applied all tests in individual sessions of 30 min each during regular school time in a private office at the same schools that the children attended.

### Intervention Program

The intervention program consisted of 1-h play sessions in 60 consecutive school days. Work sessions always comprised three phases (Table 1): (a) an initial activity (5 min) focused on activating the participants through singing and dancing, (b) a collective game designed to improve one of the three main EFs components (30 min), and (c) a closing activity (10 min) focused on metacognition that included some of the principles of mindfulness methodology (Table 2).

**TABLE 1:** Sessions’ game structure.

**Time**	**Duration**	**Content**
1	5 min	Activate and positive attitude
2	30 min	Game development
3	10 min	Metacognitive activity based on mindfulness

**TABLE 2:** Examples of initial and closing activities.

**Initial activities**	**Closing activities**
Frog family: The mediator sings the song of the frog family, making some movements to represented it. Children repeat the song and the movements. The song represents different family members using the characteristic movements of each: dad, mom, son, daughter, and baby.	Balloon inflating: Children stand in front of the mediator. They are asked to stand upright and put their hands on their bellies. Then they are told to imagine that their bellies became balloons and that they will inflate them slowly, inspiring through their noses. They are asked to pay attention to the way their bellies expand when the air enters. Then they deflate the balloon slowly.

A total of 32 different games (see Supplementary Appendix 1) were designed or adapted from existing games by the research team. Every game was specifically designed to enhance one of the three components of EFs, although most of them could also help enhance the other components. The games were gradually implemented during the program implementation, according to their cognitive demands. They were always played during the first period (length: 45 min) and were mediated by two professionals from the Center for the Development of Inclusive Technologies (CEDETi UC), which is part of the Pontificia Universidad Católica de Chile. The four participating teachers were also invited to help with the game coordination, but they devoted their game time mostly to attending to other duties in the classroom.

**FIGURE 3:**
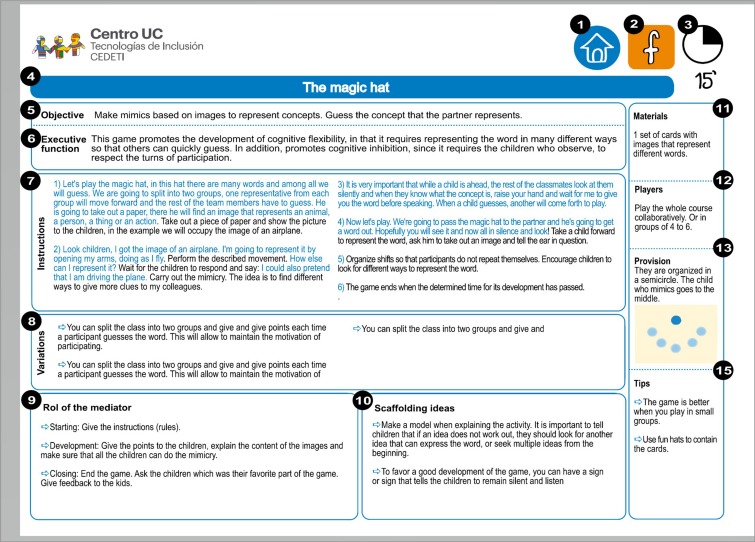
Sample of a homogenization sheet. (1) Type of game: playground, expression, or classroom games. This is a classroom game. (2) Main EFs component developed. (3) Approximate duration of the game. (4) Title of the game. (5) Aim of the game. (6) Way in which executive functions are developed. (7) Instructions. (8) Ways in which the game can be modified. (9) Role of the mediator in each phase of the activity. (10) Scaffolding ideas. (11) Materials needed to conduct the activity. (12) Number of players and organization. (13) Suggested spatial arrangement. (14) Additional suggestions.

To homogenize the intervention among the mediators, fact sheets were created by the research team for each game. These sheets referred to formal aspects such as the goal of the game, instructions, duration, spatial arrangement (classroom or playground), number of players, and materials needed, along with didactic aspects such as the mediator’s role, scaffolding ideas, possible variations, and specific advice regarding the contents of each game. Figure 3 presents a sample sheet for one of the games.

Although the focus of each session was on the game designed to develop EFs, the initial and closing activities were also aimed for the intervention program in general. In Table 2 can be seen some examples of initial and closing activities.

The participating classes had 35 children on average; however, not all parents signed the informed consents, which resulted in different numbers in the data for the experimental and control groups (experimental class 1, *n* = 29; experimental class 2, *n* = 8; control class 1, *n* = *2*2, control class 2, *n* = 11). Regarding the effective playing time, the experimental groups had on average 81% attendance during the game sessions.

Meanwhile, games were played by the two experimental groups; the children from the control groups received their traditional learning activities. For the traditional Chilean curriculum, this means that children who did not participate in the intervention (control) had personal and social development (i.e., identity and autonomy, coworking and citizenship, corporality and motor aspects), integral communication (i.e., verbal language, artistic language), and finally, interaction and environment comprehension (i.e., wild environment exploration, sociocultural context comprehension, and mathematical thinking).

### Analytical Plan

The data analysis consists in two parts. In the first, we analyze the differences between the groups, with the aim to assess the impact of the program over the development of executive functions (EFs). The differences were analyzed through a covariance analysis of the differences in the growth deltas observed in the two groups. In the second part we focus on the effects of EFs development over academic performance. These effects were calculated by doing a regression analysis using the executive function level at time 1 (T1) as a predictor over the academic performance of children in language and math at time 3 (T3).

### Results

**FIGURE 4:**
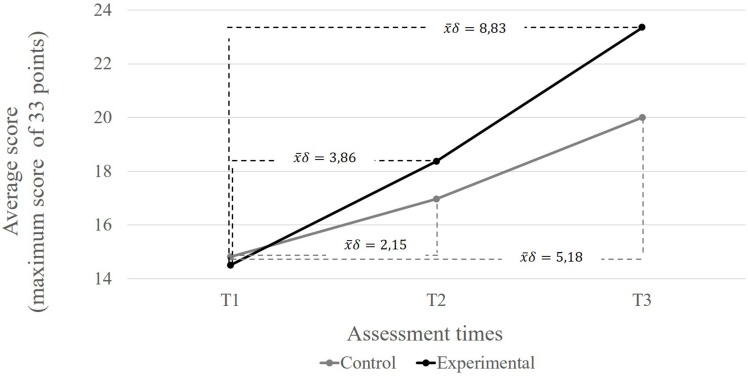
Average growth deltas observed in each group at each assessment time.

Differences in EFs performance were measured between T1 and T2 and between T1 and T3 for the experimental and control groups. The total score for phase 3 (mixed congruent and incongruent trials) is considered to be an indicator of EFs general performance. The results are shown in Figure 4 and Table 3.

**TABLE 3:** Medias, standard deviations, and percent correct outcomes of each group at different assessment moments.

	**Time 1**			**Time 2**			**Time 3**		
			
	**M**	**SD**	**PC**	**M**	**SD**	**PC**	**M**	**SD**	**PC**
Experimental	14.51	5.63	44.0%	18.38	6.0	55.7%	23.35	5.91	70.5%
Control	14.82	6.67	44.9%	16.97	7.43	51.4%	20.0	7.86	60.6%

*M, media on EFs results; SD, standard deviation; PC, percent of correct outcomes on EFs.*

Afterward, two analyses of variance were performed to examine the EFs performance between the experimental and the control group. The first analysis was performed to detect the performance differences between T1 and T2 (Delta), while the second focused on the differences between T1 and T3 (delta), controlling for the participants’ age. The results are shown in Table 4.

**TABLE 4:** EFs performance of the experimental and the control groups, compared between T1 and T2 and between T1 and T3.

	***Df***	**F**	***P***	**η_*p*_^2^**
T1-T2 performance difference	2	1.127	0.330	0.033
T1-T3 performance difference	2	3.282	0.044^∗^	0.090

*^∗^Significant at α = 0.05*

The results of the ANOVA revealed no significant performance differences between T1 and T2 between the experimental and the control group (*p* = 0.330). However, the differences were significant between T1 and T3 (*p* = 0.044), in which the experimental group (*X* = 23.35) performed better than the control group (*X* = 20). The effect size (η*_*p*_*^2^ = 0.090) was small (Cohen, 1988), and the statistical power was 1–β = 0.71.

The differences in mathematics and language performance between the experimental and the control group were compared at T3, controlling for EFs level at T1 (it is impossible to control for language and math outcomes at T1 because there is no formal instruction of these contents in the Chilean preschool system, and therefore, they were not assessed). The results revealed no significant differences in the language area; however, they were significantly different in mathematics, showing a significantly better performance for the experimental group (Table 5).

**TABLE 5:** Comparison of mathematics and language performance between the experimental and the control group at T3, controlling for EFs performance at T1.

	***df***	***F***	***p***	**η*_*p*_*^2^**
Mathematics performance	2	8.252	0.001^∗^	0.222
Language performance	2	0.771	0.467	0.025

*^∗^Significant at α = 0.001.*

Afterward, to understand the association between EFs and academic performance, we analyzed the predictive power of the Hearts and Flowers score at T1 with regard to mathematics and language performance at T3. Age for experimental and control groups was initially controlled (step 1). Then we included the T1 Hearts and Flowers performance measure (step 2). Separate regressions were generated for the standardized mathematics (Table 6) and language scores (Table 7).

**TABLE 6:** Stepwise regression for mathematics performance.

**Step**	**Variable**	**β**	**Δ*R*^2^**	***df***	***t***	***p***
Step 1	Age (months)	0.139	0.003	1	1.08	0.285
Step 2	EFs time 1	0.455	0.193	1	3.863	0.000^∗∗^

*^∗∗^Significant at α < 0.001.*

**TABLE 7:** Stepwise regression for language performance.

**Step**	**Variable**	**β**	**Δ*R*^2^**	***df***	***t***	***p***
Step 1	Age (months)	0.098	0.010	1	0.746	0.459
Step 2	EFs time 1	0.160	0.025	1	1.206	0.233

The results clearly showed that, after controlling for age, the EFs measure significantly predicted the variance in mathematics performance (0.193, *p* = 0.000) but did not have any predictive value in language performance (0.025, *p* = 0.233). These results are consistent with the ANCOVA that compared the differences between the experimental and the control group in math and language in T3 after controlling for the performance of EFs (Table 4).

Finally, we compared the students’ performance in EFs at T3 according to their initial outcomes. The experimental group sample was subdivided into three subgroups according to their performance in the EFs assessment at T1. We divided the groups at percentiles 33 and 66, thereby forming the three subgroups. Then, through an analysis of variance, we compared the growth deltas of the poorest-performing third (*M* = 10.33; *SD* = 6.41) and the best-performing third (*M* = 5.54; *SD* = 5.41). Although the results showed no significant differences between the growth deltas of the two groups, they were clearly at the limit (*F* = 4.1; *p* = 0.055), which were higher in the group with the poorest initial performance.

## Discussion

This study aimed to analyze the impact of a game-based intervention on the development of EFs in preschoolers. As described by other authors (Hermida et al., 2015; Traverso et al., 2015), the implementation of the program had a positive impact on the improvement of the participants’ EFs, which provides support for the use of such programs in preschool classrooms.

We will organize our discussion around some of the conclusions advanced by Diamond and Ling (2016) since we consider them to be essential for analyzing the causes of the program’s success.

First, regarding transference, we sought to align our program with the authors’ views: it includes a variety of games that, apart from involving physical activity, require the combined use of a number of cognitive skills. For instance, ball war not only involves picking up and throwing balls around, as children must also make a cognitive effort to identify the facial expression drawn on each ball and then decide to either throw or keep it. The games used were varied and were repeated only three times at most. This prevented the children from predicting their contents and putting less effort into them. In addition, the types of tasks used for evaluating EFs sharply differ from the games implemented in the intervention, which makes it possible to rule out the effect of direct or excessively specific training of EFs components.

One open question related to transference that was not addressed by our project is whether there can be design-specific EFs component interventions to specific EFs outcomes. As we only took a general measure of EFs, we cannot show any data in this direction, but future research should address the contradictory evidence from almost all of the studies reported regarding these issues.

Second, regarding duration, the present program attempted to greatly surpass the 32 sessions used in the study conducted by Hermida et al. (2015), who found this number to be insufficient. The participants played for 45 min per day over a 3-month period. This resulted in a total of 60 game sessions. Compared with other programs (e.g., Traverso et al., 2015), this implementation time is long; however, it is shorter than that reported for curricular programs such as “Tools of the Mind.” Implementing a game-based program such as ours at the curriculum level could have a more lasting impact on students’ EFs development. This should be tested in future studies that incorporate games over a longer period and that are able to conduct a longer longitudinal follow-up process. It is interesting to note that a very similar intervention program designed by Röthlisberger et al. (2011) also generated significant training effects in 60 sessions of 30-min activities. But in contrast to that experience, which was implemented in a small-group format, our intervention proved to be possible to implement in a totally natural classroom context, with groups with up to 30 children. This is a huge advantage of our design because it can possibly be transferred as a regular preschool activity, without the need to take groups of children apart.

Related to this, it should be noted that a program such as that proposed here, implemented during regular class hours, shows that it is more effective for EFs development than the “regular” classes attended by the control group. And given the proven association between EFs and mathematics performance 10 months later, it is necessary to consider the importance of conducting activities to promote EFs in the preschool curriculum.

The fact must be highlighted that our interventions were always implemented with the entire class of approximately 35 children, that is, a full group intervention. Some of the games required dividing the class into subgroups, of course. But our methodology, in contrast to other successful programs (e.g., Traverso et al., 2015), is designed to make its implementation possible in natural school settings.

In any case, it is important to highlight that the work of Traverso et al. (2015) shows significant outcomes in many measures of EFs after only twelve 30-min training sessions in a controlled setting, with robust size effects in the majority of them. Although they did not report any long-term effects, it is necessary to investigate more exhaustively whether their results are a consequence of the type of training tasks employed, the training setting, or a combination of the two.

In the same direction, it is important to note, however, that the intervention program’s optimal duration remains an open question. Hermida et al. (2015) showed very weak results in EFs after 32 sessions, but very strong results in the long-term effects over math and language outcomes one year later. We obtained similar results in math outcomes, but not in language outcomes. And our program effects over EFs are modest but significant.

Third, this study expressly controlled for the program monitors’ commitment and motivation, as suggested by Diamond and Ling (2016). The monitors were part of the research team and took part in the design of the games and the program. Therefore, they expected the program to have positive results and were committed to the success of the intervention. However, it is necessary to test the efficacy of the program in a more natural context, that is, where the implementation is in charge of the educators who work with the children.

Fourth, this program has a design that constantly challenges children’s EFs, at least for 45 min per day for over 3 months. Organizing the sessions around mindfulness activities, games, and a cognitive closing phase in which the participants metacognitively reflect on the games can also allow the children to learn a more general way to approach tasks. In this regard, this program is consistent with others in which activities are designed to permanently challenge children’s EFs (Hermida et al., 2015) but extend the intervention compared with brief programs, whose longer-term effects are unknown (Traverso et al., 2015).

Fifth, our study is consistent with what Diamond and Ling (2016) report, as we observed that individuals with lower EFs development levels tend to benefit more from programs of this type.

Although research shows that the effect of these programs fades over time, 8 months after they are finished, our program displayed a better effect than immediately after it ended. This is a promising result since it suggests that game-based strategies promote a more lasting development of EFs.

This project yielded no information about the cognitive load of tasks and their greater influence in programs aimed at developing EFs (Diamond and Ling, 2016) since we only tested EFs with the gold standard for their overall evaluation: the Hearts and Flowers task devised by Wright and Diamond (2014). Likewise, our program did not aim to generate evidence about whether physical activity by itself is a good way to foster EFs, as suggested by Hillman et al. (2018) in their response to Diamond and Ling’s (2016) criticism. Still, what we do consider relevant is to incorporate games with a major aerobic component since preschoolers are very open to and motivated by games of this type. Yet in all the games included, our program explicitly sought to develop a given EFs component; thus, we have no information that could shed light on the issue.

Lastly, it cannot be completely ruled out that our program was affected by intervening factors beyond our control. We believe that the main potential issue was that the experimental group had very motivated monitors with lengthy experience in classroom games with small children. In contrast, the control group attended to regular classes with their regular teachers. Although we made sure to select only teachers with good scores on the CLASS Pre-K^®^ scales (Pianta et al., 2008), the novelty and highly interactive nature of the games played by the experimental group could have a strong impact on the motivation of the children. Although this is a possibility, it does not negate the fact that the proposed program, after controlling for all the aspects listed by Diamond and Ling (2016), has a significant effect on the development of EFs in preschoolers, which was measured 8 months after the end of the intervention.

Also, the results of our intervention, despite its modest effect sizes, show that the suggestions laid out by Diamond and Ling (2016) give a good framework to the design and implementation of high-quality (Barnett, 2011) and replicable programs for the enhancement of EFs, with proven, lasting effects.

Future research interventions should include more variables, as IQ or sociodemographic factors that could have an impact over the program results.

## Author’s Note

RR is the PI of the Fondecyt Grant that financed this work.

## Data Availability

The datasets generated for this study are available on request to the corresponding author.

## Ethics Statement

This study was approved by the Vicerrectoría de Investigación (VRI) (Vice-President’s Office for Research) of the Pontificia Universidad Católica de Chile through the ethics committee of the Faculty of Social Sciences of the School of Psychology, thus meeting global norms for social science research. The children’s parents signed an informed consent form to authorize them to participate in this study, and the children gave their verbal assent before the start of each evaluation.

## Author Contributions

RR designed the study and wrote the final version of the manuscript. VE coordinated the research, analyzed the data, and wrote the first draft of the theoretical part of the manuscript. FP wrote the method and built the final databases for the manuscript. FC helped with the writing of the discussion.

## Conflict of Interest Statement

The authors declare that the research was conducted in the absence of any commercial or financial relationships that could be construed as a potential conflict of interest.
