# Supplementary material for: Executive Functions Can Be Improved in Preschoolers Through Systematic Playing in Educational Settings: Evidence From a Longitudinal Study
Source: Front Psychol. 2019 Sep 3;10:2024. doi: 10.3389/fpsyg.2019.02024 (PMC6734167; doi:10.3389/fpsyg.2019.02024)
Supplement: Supplementary file 1 [file Table_1.pdf]

Supplementary Appendix 1

Games included in the intervention divided by the kind of game and for executive functions' component that is mainly enhanced through the game

Inhibition games		
**Playground games**	**Expression games**	**Classroom games**
**The guardian:** Children are placed next to each other above a line about some distance from the monitor. When the monitor turns against the wall, the children should move toward it silently. And when the monitor turns to the children, they should remain motionless. The monitor walks among the children, and if he realizes that someone is moving, that person must go back to the initial place. The child who arrives first touches the mediator and wins.	**Simon says:** This is the classic game of "Simon says." The monitor tells the children a series of simple indications, such as "jump on one foot" or "put the finger on your nose," and they should only execute them when the indication is preceded by "Simon says." To increase the difficulty level, other names are added as distractors.	**Five:** A story is told to the children; this story should contain some numbers. The children should listen in silence, but when they hear the number 5, they should clap three times. To increase the difficulty, you can add different actions to be performed with different numbers.
**Ball war:** The class is divided into two teams, and a line is made on the ground. Each team is placed on one side of the line. Many small balls with happy, sad, and angry faces are distributed among the children. Happy faces give 2 points for the team, sad and angry faces take 1 point each. When the signal is given, the children should throw all the balls with sad and angry faces toward the other side and keep those with happy faces. The	**Frozen:** The children are arranged in a circle. When the music plays, the children must dance; but when it stops, they must remain motionless. To increase the difficulty level, the following rule is added: when you show them an image of a musical note, they must dance independently of what happens with the music; and when an image that represents silence is shown, they must remain frozen even if the music is played.	**Feeling nervous:** You must have 15 cards per child; these cards must have drawn quantities from 1 to 5 (always in the same arrangement). The children must hold in their hands the 15 cards, but without looking at them. In turn, they have to throw into the pile one card, repeating aloud the numbers from 1 to 5. If the number they say matches the number of elements present in the card that is being thrown to the pile, then everyone must put

team that has the most points wins. To increase the difficulty level, the rule can be changed: the happy and sad faces should be thrown to the other side, and the angry faces should be kept.		their hand on the heap. The person who puts his hand after everyone else loses. The person who loses starts the game again.
**Penalties:** The game consists of throwing penalties to the goal (kick the ball to a soccer goal). The children stand in a row, and the monitor has the balls in a bag and takes them out when the children approach so that they see them at the moment of kicking. If the ball is red, the children have to kick it; if it is blue, they have to throw it with their hands toward the goal; and if it is yellow, they have to throw the ball differently. To increase the difficulty, you can change the rules, or you can say that with a specific color, the child remains immobile.	**Stripped wires:** The monitor makes a body movement, and the children must imitate him. Then he tells them to do, and the children must do it. Then he tells them an action, but at the same time, he performs another movement (he says, "Put your right hand on the head," but he puts his left hand over his eyes). The children should follow the verbal instructions and ignore the movement the monitor makes. To increase the difficulty, after playing this way for a while, the rule is changed. The children should copy the action and ignore oral instructions.	**Interview without yes or no:** The children sit in a circle. The monitor asks them quick questions one by one, and they must respond without saying "yes" or "no." To increase the difficulty level, a stop sign is added. If the sign is shown, the children cannot say "yes" or "no"; but if the sign is hidden, they should say at least one of those words in their response.
**Ducks into the water:** Children stand around a circle demarcated on the ground. The circle represents a lake. Half of the children will be ducks and half cats. When the monitor says "into," all the ducks will jump into the circle, and the cats will jump outside. On the other hand, when he says "out of," the ducks will leave the circle, and the cats will enter. The words "out of" and "into" should be said to be		**Happy and sad:** You have to show the children a poster with happy and sad faces drawn in an orderly fashion in rows and columns. Some faces must be drawn blue and others red. The children are asked to name the faces from left to right and from top to bottom according to their expression: for example, "happy, happy, sad, happy, sad, etc." Then they are asked to name them according to color: for

messy, and the children should do what corresponds according to whether they are ducks or cats. To increase the difficulty, once played in the previous way, the groups are changed. The ducks become cats, and the cats become ducks.

example, “red, blue, blue, red, blue, etc.” You must repeat these exercises until you reach a higher speed. To increase the difficulty, the children should be asked to name one by one the face according to their color, and according to the emotion they represent: for example, “red, happy, blue, sad, blue, etc.” You can go further by inventing different rules.

Emergency call: You must have a large telephone (it can be drawn on a poster). The kids participate by turns. The monitor dictates a sequence of three numbers, and the participating child must mark it on the phone. If he does it well, the phone will be answered by a friendly character (represented by a puppet); and if he does it wrong, the phone will be answered by an unpleasant (but fun) one. To increase the difficulty level, the number of numbers in the sequence should be increased, or the children could be asked to order the dictated numbers from least to greatest or from highest to lowest.

Fishes and octopuses: There must be a series of cards with fishes and octopuses of different colors (blue, red, and yellow) and with different textures (dots and

		stripes). You must also have a dice to indicate the type of animal (with shapes of fishes and octopuses on their faces), another to indicate the color (with blue, red, and yellow colors on their faces), and another to indicate the texture (dotes or stripes). The cards have to be put faceup so that all the children can see them. The three dices are thrown. The children should look at what appears on the dice and look for the corresponding animal; for example, if the color is blue, the shape is a fish, and the texture is dotted, look for a blue dotted fish. The first child to find the animal indicated by the dices wins and takes the card.
**Working memory**		
**Playground games**	**Expression games**	**Classroom games**
**Treasure hunt:** The children should be organized in teams. To find the treasure, they must find and decode a series of clues (four or five clues) that are hidden somewhere in the schoolyard. Before reading the clues, they must decode a three-digit code. This means that the children order a series of numbers that are told in a disorderly way from highest to lowest. Then the monitor reads the track that contains a series of steps with the indications to get to the next	**War of rhythms:** The children must be separated into two groups. The monitor exemplifies a percussion with applauses. The first group of children must copy it, and the others must remain silent. Then another percussion is performed, and children from group 2 must perform it, while those of from the first group remain silent. Subsequently, the different groups must perform their percussions interspersed without stopping. Once one group ends, the other begins.	**The train:** The monitor gives a category to the children, and they have to say elements that belong to that category without repeating them. For example, the monitor says, “The train comes charged with animals, such as horses.” The children should say animals without repeating. When someone repeats or cannot think of anything, they start again with a new category.

place. The children should follow the directions until they reach the place where the treasure is hidden. To increase the difficulty level, the number of digits of the code and the number of instructions in each clue should be increased.	They must do it faster and faster until one group fails. Then they start again using a different percussion. To increase the difficulty, the rhythmic sequences must be longer.	
**Crazy race:** The field of play is divided into three zones using lines or other elements to demarcate. The children are placed in three or five rows with an equal number of participants. The first ones from each row start the race. They must go jumping in two feet like kangaroos until the first line, soon moving their arms like birds until the second line, and then they have to walk in four legs (with arms and legs) like dogs until the fourth line. When they reach the end, they must return running and touch the hand of the next child in their line, who runs the same circuit. The team that finishes first wins. To increase the difficulty level, instead of returning running, the children return performing three other actions.	**Twister game:** Each group (five children) is placed in front of a board of the traditional game twister (six rows and four columns of circles with four different colors). The children line up in front of the board. The monitor says a series of four colors (e.g., blue, yellow, red, yellow), and the children must go to the other side by jumping on the circles with different colors following the sequence. They take turns doing it. To increase the difficulty, the monitor can increase the number of colors in the sequence or say sequences that involve putting certain body parts in specific colors, as in the traditional game.	**Crazy costumes:** There must be a set of costumes and accessories. One of the children is placed at the center. In turn, three classmates each puts on an accessory: for example, the first child puts on a cloak, the second puts on a hat, and the third puts on a pair of glasses. Then another student must get ahead and remove the accessories in reverse order in which they were placed. In this case, the monitor should first remove the glasses, and then the hat, and finally the coat. To increase the difficulty, he can add more accessories to the participants.
	**Fun dance:** The children are arranged in a circle. Before playing the music, the dance steps are defined: when the mediator says "A," the children will raise and lower their arms;	**Memory lottery:** Each child receives a bingo card with six blank spaces and six figures with different shapes and colors. The monitor has the same materials, but larger. For 5 seconds, he

	when he says "E," the children will jump; when he says "O," the children will move their arms from side to side; and when he says "U," the children will applaud. The music is played in a moderate volume, and the mediator tells the sounds. In the beginning, he models the steps, but then the children perform them alone. After a while, the mediator can change some of the movements but keeps the sound. For example, when he says "O," the children will dance with their hands on their waists. To increase the difficulty, a sequence of sounds can be said in the beginning, and then the children must perform the movements when the music is played.	shows to the children the sample cardboard with a figure located in a specific space of the bingo card. Then the children should copy it in their own bingo card. The difficulty increases by including more figures in different positions.

**Cognitive flexibility**		
**Playground games**	**Expression games**	**Classroom games**
**Body shapes:** The children are separated into groups of five. The monitor says the shape that the children must represent using their bodies. For example, "a car." Between all, they must form a car. One of the children can be the handlebar, two children can be the wheels, and two can be parts of the body of the car. Then they are asked to form a car but have another form. They are asked for different proposals until	**Sing along:** The monitor needs to use a song well-known among the children, for example, "Trinkle Twinkle Little Star." First, he sings with the children the song in the traditional way. Then the children are encouraged to change the lyrics of the song but keep the melody. Each child should say a sentence but considering the context so that the new song makes sense. Once a song ends, another one can be	**Story cubes:** The monitor must have four dices that have images on their faces (like the dices in the "story cubes" game). It is played in groups of five children. In turns, they roll a dice, and they must tell a story. For example, if a flower appears, the child can say, "There was once a flower." Then the next child rolls a dice and continues the story according to the image that appears. For example, if a sun appears, the

they cannot think of anything else or until they are too similar. They should also be fixed in the forms proposed by their peers from other groups.	invented using the same melody, but with another theme.	next child can say, “That flower grew under a vast sun.” And so a story that makes sense continues.
**Labyrinths:** With different colored adhesive tapes, disordered lines are marked on the floor (or they can be the lines drawn in a school court). The children are asked to go from one side of the “labyrinth” to another, taking turns. Each child must travel the road from one point to another following the lines, without repeating the path made by his previous classmate. In this way, they should look for different ways to get to the other side following the different paths that can be followed above the lines.	**Just dance:** The children are taught specific dance steps; each dance step must be associated with an animal. For example, “the step of the duck” will be picking up the arms and moving them up and down, the step of the kangaroo “will be jumping,” and so on. The children should do simple dance sequences, for example, “kangaroo, duck, rabbit, duck.” Then the rule must be changed; the duck will be the kangaroo, and the kangaroo the duck. They must reproduce the dance sequences previously practiced, but with the instructions reversed.	**Who am I?:** Cards with characters are distributed to the children (animals, professions, tale characters, etc.). Each one puts the card on his forehead, without looking at it, so that everyone knows which card the others have, but they do not know their own. By turns, everyone should ask a question about their character to guess who it is; others can only answer “yes” or “no.” For example, “Am I an animal? Do I have hair?” The person who guesses his character first wins. The game can be played until everyone guesses.
		**Memory game:** You must have cards that have elements that belong to different categories: for example, six cards with animals, six cards with sports, six cards with clothing, and so on. All the cards are placed facedown in a disorderly manner. By turns, the children must turn two cards. If both cards belong to the same category, the player stays with that pair. If they correspond to different categories, the player must return them to the place

where they were. Whoever gathers more cards at the end of the game wins.

Different uses of . . . : The monitor must have different objects in a bag. An object is shown to the children, and they are asked what it is use for. The children must answer saying various forms of use of the object, but without repeating. They can even invent unconventional ways in which the object can be used. The game continues until the ideas are repeated or until there are no more ideas. In that case, it starts again using a new object.

Let's build a . . . : There must be different Lego pieces to distribute to groups of children. The children will be asked to build a specific object, for example, a house. Once they finish, they will be asked to build another house, but it has to be different from the first one. And then they will be asked to build a third house, but it has to be different from the first and second ones. Then they will be asked to perform other elements.

Drawings in circles: The children are divided into groups of five. Each group receives a paper with at least 20 circles. The children are asked to make

	different drawings using the circles as a base. The team that makes the most drawings using the circles wins.<br>**The magic hat:** Children are separated into groups of 10. Each group has a hat with cards with different images (but it has to be the same set for each group). By turns, they must get ahead, take a card, and represent it with mimics. The other children must guess. Once the game is over, all the groups come together, and the children who represented the same word are asked to repeat their mimicry. Together they see the different ways in which the same word can be represented and think of other ways of using it.
